# Permeation of Insulin, Calcitonin and Exenatide across Caco-2 Monolayers: Measurement Using a Rapid, 3-Day System

**DOI:** 10.1371/journal.pone.0057136

**Published:** 2013-02-27

**Authors:** Vivek Gupta, Nishit Doshi, Samir Mitragotri

**Affiliations:** Department of Chemical Engineering, University of California Santa Barbara, Santa Barbara, California, United States of America; Glasgow University, United Kingdom

## Abstract

**Objectives:**

Caco-2 monolayers are one of the most widely used *in vitro* models for prediction of intestinal permeability of therapeutic molecules. However, the conventional Caco-2 monolayer model has several drawbacks including labor-intensive culture process, unphysiological growth conditions, lack of reproducibility and limited throughput. Here, we report on the use of 3-day Caco-2 monolayers for assessing permeability of polypeptide drugs.

**Methods:**

The 3-day monolayers were grown in a commercially available transwell set-up, which facilitates rapid development of the Caco-2 monolayers in an intestinal epithelial differentiation mimicking environment. This set-up included use of serum-free medium of defined composition with supplements such as butyric acid, hormones, growth factors, and other metabolites, reported to regulate the differentiation of intestinal epithelial cells *in vivo*. We measured permeability of 3 different therapeutic polypeptides; insulin, calcitonin, and exenatide across the monolayer.

**Results:**

Preliminary validation of the monolayer was carried out by confirming dose-dependent permeation of FITC-insulin and sulforhodamine-B. Transport of insulin, calcitonin, and exenatide measured at different loading concentrations suggests that the permeability values obtained with 3-day cultures resemble more closely the values obtained with *ex vivo* models compared to permeability values obtained with conventional 21-day cultures.

**Conclusions:**

Short-term 3-day Caco-2 monolayers provide new opportunities for developing reproducible and high-throughput models for screening of therapeutic macromolecules for oral absorption.

## Introduction

Macromolecular drugs including therapeutic peptides and biologicals such as insulin and vaccines are the preferred therapies for various systemic diseases 1]. However, owing to their proteolytic degradation in the gastrointestinal tract and poor permeation across the intestinal epithelium, all therapeutic macromolecules suffer from poor oral bioavailability and thus have to be administered via parenteral route 2,3]. Parenteral route, though effective in administration of therapeutics, suffers from severe limitations including pain, needle-phobia and poor patient compliance. Hence, significant attention is being dedicated to the development of orally administered formulations of biologics 4–7]. Owing to the challenges and complexity of *in vivo* models of oral absorption, *in vitro* experimental models that allow assessment and/or prediction of oral bioavailability play a vital role in development of oral biologics.

Several experimental models have been developed for predicting intestinal permeability including *in situ* isolated perfused intestinal systems, everted gut sac and cultured cell monolayers 8]. Among these, Caco-2 cell monolayers are one of the most studied approaches for predicting oral absorption of therapeutic peptides, and are considered the gold standard for predicting *in vitro* intestinal permeability and absorption 9–12]. Caco-2 is a human colon carcinoma cell line, which when grown on permeable filter supports, grows into monolayers with differentiated phenotypes exhibiting many salient features of small intestinal villus epithelium, the most important ones being development of intercellular tight junctions and inclusion of various metabolic enzymes present in the intestinal epithelium. Caco-2 cell lines have been widely used to predict oral absorption of novel therapeutic agents during early stages of development.

A conventional fully differentiated and confluent Caco-2 monolayer development requires about 3 weeks to culture with 9–10 labor-intensive cell feedings, accounting for a significant lead time between experiments, thus limiting the throughput. At the same time, the conventional 21-day Caco-2 monolayers are reported to develop unphysiologically tight junctions (TEER values ∼300 Ω.cm^2^), compared to human small or large intestine *in vitro* (TEER ∼50–100 Ω.cm^2^) 13,14]. Concurrently, traditional Caco-2 cultures are performed with undefined animal serum, which accounts for a significant variability among results from different laboratories. Keeping the limitations of 21-day Caco-2 culture in mind, several groups have investigated the possibilities of developing a more rapid Caco-2 culture mimicking the intestinal epithelial differentiation environment with (i) reduced serum requirements 15], or (ii) a serum-free 3-day short-term Caco-2 culture 13]. Both these systems have been tested for efficacy in providing reproducible permeability measurements. The 3-day Caco-2 system however: (i) provides physiologically relevant tight junctions (TEER ∼50–100 Ω.cm^2^) 13], (ii) expresses similar levels of different metabolic enzymes such as brush border peptidase and alkaline phosphatase, and functional transporter (P-glycoprotein) activity 16], and (iii) is suitable for increased throughput 17].

A variety of small molecules have been tested in 3-day cultures and compared with their evaluation in 21-day cultures 13,17]. The use of 3-day Caco-2 cultures for evaluating macromolecules, on the other hand, has been rarely reported. As mentioned earlier, therapeutic peptides are some of the most challenging molecules for developing oral formulations. However, there are large differences among the reported intestinal permeability values for therapeutic peptides, which make it difficult to predict the course of oral absorption of a specific peptide based on available data, which are reported to have negligible oral bioavailability. Here, we report on the use of serum-free 3-day Caco-2 cultures for assessing permeation of three therapeutic peptides: bovine insulin, salmon calcitonin and exenatide (exendin-4 analog).

## Materials and Methods

### Materials

FITC-labeled bovine insulin, sulforhodamine B, and bovine insulin were obtained from Sigma Aldrich (St. Louis, MO, USA). Salmon Calcitonin was obtained from Anaspec, Inc. (Fremont, CA, USA). Exenatide (Exendin-4) was obtained from Tocris Biosciences (Minneapolis, MN, USA). The transwell Caco-2 system was set up by using 24 well BD-Biocoat™ HTS Caco-2 assay system (fibrillar collagen coated, 1 µm pore size) obtained from BD Biosciences (Bedford, MA, USA). ELISA kits for analysis of different peptides were obtained from various commercial vendors. Bovine insulin ELISA kit was obtained from Mercodia, Inc. (Winston Salem, NC, USA). Extraction-free salmon Calcitonin and exenatide ELISA kits were obtained from Bachem Americas, Inc. (Torrance, CA, USA). Supplies for Caco-2 culture were obtained from Fisher Scientific (Pittsburgh, PA, USA). All the ELISA kits used for the quantification analyses were peptide-specific, and were unlikely to pick any cross-reactive peptide and/or degraded peptide fragments.

### Caco-2 Cell Culture

Human colorectal adenocarcinoma Caco-2 cell line (HTB-37) obtained from American Type culture Collection (ATCC) (Manassas, VA, USA) was used for all experiments. Cell lines were maintained as per the provider's protocol. Cell culture was performed in Hyclone^®^ DMEM high glucose medium (Thermo Fisher Scientific, Pittsburgh, PA, USA) supplemented with 50 IU/ml of penicillin, 50 mg/L of streptomycin, and 100 ml/L of fetal bovine serum at 37°C in a humidity-controlled 5% CO_2_ cell culture incubator. Cells were split at a ratio of 1∶3 after reaching 90% confluence. All transwell experiments were performed with cells between 5^th^–14^th^ passages due to possible phenotypic differences between cells from high and low passage intervals. Cells were cultivated for at least 2 passages before seeding onto transwell filter supports to stabilize the cell phenotype 12].

### Transwell Assay System Set-up

Transwell experiments were performed with slight modifications to the protocols provided by the manufacturer (BD Biosciences, Bedford, MA, USA). For the development of the transwell assay system for permeability experiments, Caco-2 cells were seeded onto fibrillar collagen coated polyethylene terephthalate (PET) filter supports (1 µm pore size) of BD Biocoat™ HTS Caco-2 assay system (BD Biosciences, Bedford, MA, USA) according to manufacturer's protocol with slight modifications. Briefly, Caco-2 cells were seeded onto the filter supports at a concentration of 200,000 cells/insert (6.6×10^5^ cells/cm^2^) using basal seeding medium (with MITO+ serum extender) provided in the HTS system, and were incubated in cell culture incubator (37°C, 5% CO_2_). Cells were fed from both sides using the Multiwell feeder tray. After 24 hrs, the feeding medium was replaced with MITO+ serum extender supplemented Entero-STIM medium, and growing monolayers were incubated as described earlier. Upon incubation for 48–72 hrs, Caco-2 cells developed a tight junction monolayer, integrity of which was determined by TEER measurements.

MITO + serum extender used in this study is lyophilized from a solution of Dulbecco's Phosphate Buffered Saline (DPBS) containing ECGS, EGF, insulin, human transferrin, triiodothyronine, progesterone, estradiol-17β, testosterone, hydrocortisone, selenium, and o-phosphorylethanolamine; and is reconstituted in 5 ml dH2O (stock solution). Entero-STIM is a serum-free defined medium (DMEM) containing butyric acid. Butyric acid induces differentiation of intestinal epithelial cells in vitro (by down-regulating c-myc expression) 18].

### Transepithelial Electrical Resistance (TEER) Measurements

The integrity of Caco-2 monolayer was determined by measuring the transepithelial electrical resistance (TEER) of the cell monolayer grown on filter supports using Millicell-ERS electrical resistance measuring system (Millipore, Bedford, MA) using chopstick electrodes. Briefly, the Caco-2 inserts were transferred to a 24-well culture plate with 1400 µl medium in the feeding well, and 500 µl in culture inserts. The electrodes were immersed in a way that shorter electrode was in the insert and longer electrode in the outer well. Care was taken that the electrode did not touch the monolayer. Based on the literature, a resistance reading of 150–200 Ω.cm^2^ was considered as indicative of a confluent Caco-2 monolayer with tight junctions.

Precautions were while taking TEER measurements throughout the experiment to avoid cross contamination and loss of Active Pharmaceutical Ingredient (API). The electrodes of the TEER probe were thoroughly rinsed with 70% ethanol before the start of each experiment, and also after each measurement from each individual well. At the same time, the probe was gently tapped to the side walls of the transwells to avoid any possible loss of API. For all the samples withdrawn from the basolateral side, equivalent amount of fresh release medium was added, and was accounted for while calculating cumulative transport.

### Validation of Transwell System using FITC-Insulin and Sulforhodamine B Transport

Use of transwell system in determining transport of active molecules across Caco-2 monolayer was validated by using two fluorescent molecules, FITC-insulin and sulforhodamine-B, which would delineate the efficacy of system for both macromolecular and small molecular weight pharmaceutical moieties. Briefly, cells were pre-conditioned with basal seeding medium for 30 minutes before starting the experiment at 37°C. FITC-insulin and sulforhodamine-B were loaded onto the individual Caco-2 monolayer filter supports at various concentrations (0.05, 0.15, 0.3, and 0.6 mg/well) dissolved in 500 µl of basal seeding medium. The basolateral chamber consisted 1400 µl of the same growth medium as per manufacturer's protocol. The plates were incubated for 5 hrs at room temperature with gentle shaking (5 rocks/minute on a rocker, so as to mimic intestinal peristaltic movement). TEER measurements were performed at predetermined time intervals (0. 0.25, 0.5, 1, 2, 3, and 5 hrs). At the same time-points, 100 µl samples were withdrawn from the basolateral chamber to quantify the total amount of FITC-ins/sulforhodamine-B transported across the monolayer. The withdrawn sample was immediately replaced with equivalent amount of the experimental medium. Withdrawn samples were analyzed using a Tecan Saffire™ fluorescent microplate reader (Tecan Group Ltd, Mannedorf, Switzerland) at respective wavelengths for FITC-insulin (Ex 488 nm; Em 525 nm) and sulforhodamine-B (Ex 560 nm and Em 590 nm).

### Polypeptide Transport across Caco-2 Monolayer

Transport of three different macromolecular pharmaceutical peptides was studied across the Caco-2 monolayers at different loading concentrations. Three different polypeptides, bovine insulin, salmon Calcitonin, and exenatide (exendin-4) were chosen for this study. Briefly, the polypeptides were loaded individually in apical chambers at various loading concentrations; bovine insulin (0.05, 0.15, 0.6, and 1 mg/well), salmon Calcitonin (5, and 24 µg/well), and exenatide (0.3, 1, 3, and 9 µg/well). Different doses were selected for different polypeptides based upon the values reported in literature to not only approach therapeutic efficacy but also to provide a valid comparison with the results obtained in 21-day monolayer system. Polypeptides were incubated with Caco-2 monolayers for 5 hrs at room temperature with gentle shaking. TEER measurements were performed and samples were collected from basolateral chambers of the Caco-2 plates to determine total drug transport and apparent permeability at different concentrations. All three polypeptides were analyzed with their specific ELISA kits; bovine insulin (Mercodia, Inc., Winston Salem, NC, USA), calcitonin and exenatide (extraction-free ELISA kits, Bachem Americas, Inc., Torrance, CA, USA).

### Determination of Apparent Permeability (P_app_)

The apparent permeability coefficients (P_app_) of all polypeptides were calculated using the following equation 19]:
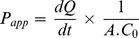
(1)


Where *dQ/dt* is the amount of solutes transported across the Caco-2 barrier in time *dt*, *C_0_* is the solute concentration in apical compartment at time zero, and *A* is the cross-sectional area of the epithelium in contact with apical solution. Total percent drug transport was calculated by dividing the cumulative amount of molecules transported with the original loading concentrations.

### Data Processing and Statistical Analysis

The data generated from *in vitro* Caco-2 transwell studies were processed using Microsoft Excel (Microsoft, Inc., Redmond, WA), and GraphPad Prism version 5.0 (GraphPad Software, La Jolla, CA). All the data have been presented in terms of mean±SD of 3 individual experiments in triplicates (n = 3). Statistical differences among the groups were analyzed by student's t-test and/or one-way Analysis of Variance (ANOVA) followed by appropriate *post hoc* analysis. Values showing *p*<0.05 were considered significantly different.

## Results

### Dose-dependent Transport of FITC-insulin and Sulforhodamine-B across Caco-2 Monolayers

**Figure 1:**
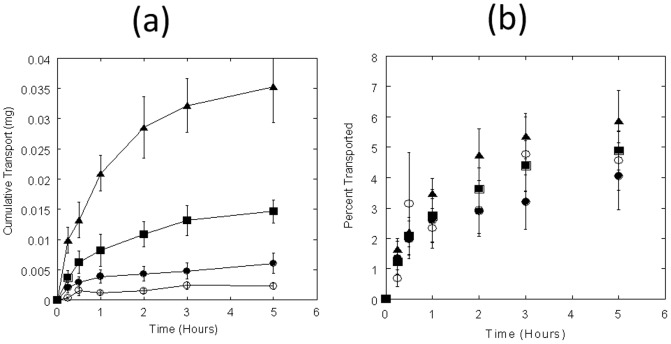
FITC-insulin transport across Caco-2 monolayers. (**a**) Time-course study of FITC-insulin transport (mg) at different loading concentrations. FITC-insulin was loaded in apical chambers at 0.05 (open circles), 0.15 (filled circles), 0.3 (squares), and 0.6 (triangles) mg/well respectively; and permeation was measured by measuring the fluorescence in samples collected from basolateral chamber at different time-points up to 5 hrs. (**b**) % FITC-insulin transport across Caco-2 monolayers. Data represent mean±SD (n = 3).

Before testing the transport of therapeutic peptides, the 3-day Caco-2 monolayers were validated by studying the permeation of fluorescein isothiocynate conjugated bovine insulin (FITC-insulin) and sulforhodamine-B. Only small quantities of the FITC-insulin permeated from the apical chamber to the basolateral chamber (Fig. 1). Transport of FITC-insulin was dose-dependent (*r* ^2^ = 0.99) in flux as well as cumulative transport. The transported amounts were: 0.002±0.0004 mg (0.05 mg loading), 0.006±0.001 mg (0.15 mg loading), 0.02±0.002 mg (0.3 mg loading), and 0.04±0.006 mg (0.6 mg loading) after 5 hours (Fig. 1a). The apparent permeability coefficients (P_app_) calculated from cumulative permeability data ranged from 8.2±1.8×10^−6^ cm/s to 10.5±1.8×10^−6^ cm/s for the loading studied here (Table 1). Cumulative transport of FITC-insulin at the end of 5 hours for FITC-insulin ranged from 4.1±1.1% (0.15 mg loading) to 5.9±1.0% (0.6 mg loading) (Fig. 1b; Table 1).

**Table 1:** Permeability values under various conditions tested in this study.

Molecule	Apical Loading (mg)	Apparent Permeability (P_app_), 10^−6^ cm/s	% Transport in 5 hours
FITC-Insulin	0.05	8.2±1.8	4.6±1.0
	0.15	7.3±2.0	4.1±1.1
	0.3	8.8±1.1	4.9±0.6
	0.6	10.5±1.8	5.9±1.0
Sulforhodamine-B	0.05	5.0±2.9	2.8±1.6
	0.15	4.9±0.9	2.7±0.5
	0.3	5.3±0.8	2.9±0.4
	0.6	4.0±0.6	2.3±0.4
Bovine Insulin	0.05	5.4±2.9	3.0±1.6
	0.15	4.2±0.9	2.3±0.5
	0.3	4.0±1.2	2.3±0.7
	1.0	4.5±0.9	2.5±0.5
Salmon Calcitonin	0.005	2.0±0.07	1.1±0.04
	0.024	1.5±0.7	0.8±0.4
Exenatide	0.0003	7.8±0.9	4.3±0.5
	0.001	5.9±2.3	3.3±1.3
	0.003	3.1±2.0	1.7±1.1
	0.009	4.2±2.1	2.4±1.2

Data represent mean±SD (n = 3).

**Figure 2:**
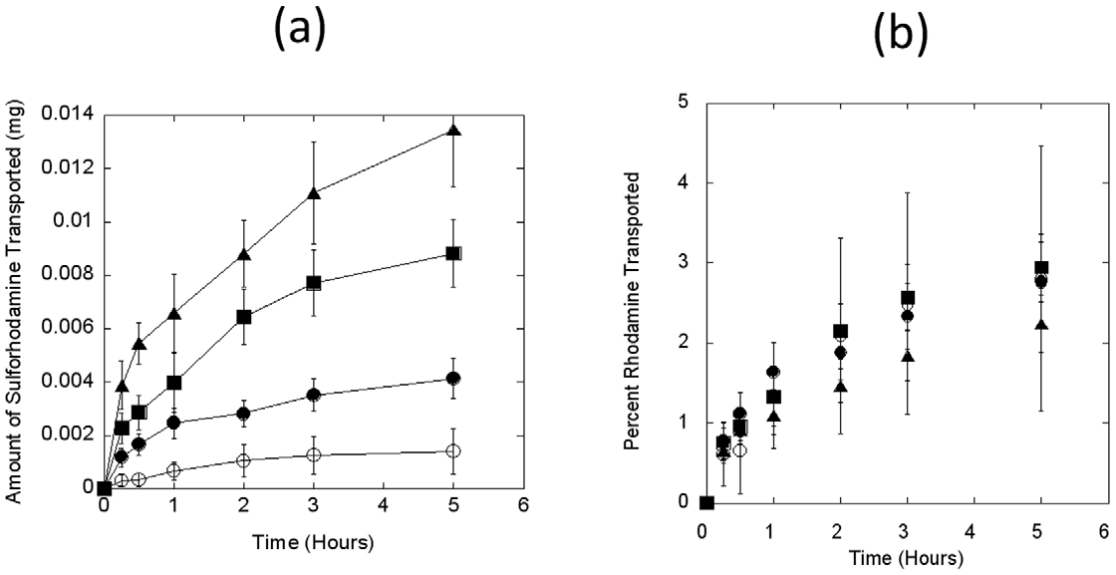
Sulforhodamine-B transport across Caco-2 monolayers. (**a**) Time-course study of sulforhodamine-B transport (mg) at different loading concentrations. Sulforhodamine-B was loaded in apical chambers at 0.05 (open circles), 0.15 (filled circles), 0.3 (squares), and 0.6 (triangles) mg/well respectively; and apical-to-basolateral permeation was measured by measuring the fluorescence in samples collected from basolateral chamber at different time-points up to 5 hrs. (**b**) % Sulforhodamine-B transport across Caco-2 monolayers over of 5 hrs of incubation. Data represent mean±SD (n = 3).

Transport of sulforhodamine-B also exhibited similar trends as FITC-insulin. Once again, a low percentage of applied sulforhodamine-B permeated through Caco-2 monolayer. The cumulative apical-to-basolateral transport of 0.002±0.0008 mg, 0.004±0.0007 mg, 0.009±0.001 mg, and 0.01±0.002 mg was observed at apical loadings of 0.05, 0.15, 0.3, and 0.6 mg/well at the end of 5 hours (*r* ^2^ = 0.977; Fig. 2a). The cumulative transport ranged between 2.2±0.4% (0.6 mg loading) to 2.9±0.4% (0.3 mg loading) (Fig. 2b). At the same time, a consistent P_app_ was also observed for all loading concentrations in the range of 4.0±0.6×10^−6^ cm/s to 5.3±0.8×10^−6^ cm/s (Table 1).

### Dose-dependent Transport of Macromolecular Peptides across Caco-2 Monolayers

**Figure 3:**
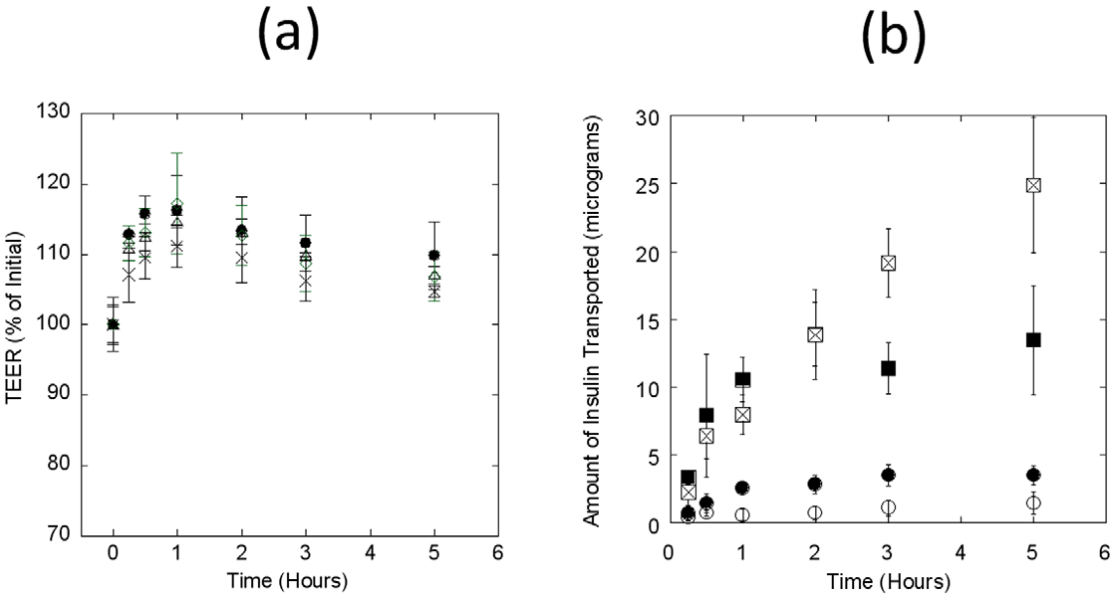
Bovine insulin transport across short-term 3-day Caco-2 monolayers. Transport of active peptides was measured across the confluent Caco-2 monolayers at different loading concentrations. 0.05, 0.15, 0.6, and 1.0 mg of bovine insulin was loaded onto Caco-2 monolayer in apical chambers by dissolving the same in the culture medium. Apical-to-basolateral permeability was determined by analyzing the samples collected from basolateral chambers at different time points for up to 5 hrs. TEER values were also measured to account for the integrity of the monolayer during the experiment. Bovine insulin concentrations were measured by commercially available ELISA kits, as mentioned in the Methods section. (**a**) TEER values of Caco-2 monolayer at different loading concentrations of 0.05 (circles), 0.15 (triangles), 0.6 (diamonds), and 1.0 (crosses) mg/well of bovine insulin. (**b**) Cumulative amount of insulin transported (µg) to the basolateral chamber at different time-points at different loading concentrations of 0.05 (open circles), 0.15 (filled circles), 0.6 (filled squares), and 1.0 (open squares) mg/well. Data represent mean±SD (n = 3).

Permeation of three different polypeptides, bovine insulin, salmon calcitonin, and exenatide (exendin-4) across Caco-2 monolayers was studied. The TEER values did not exhibit a drop during the course of the experiment, indicating that the cell monolayer was intact and the transport of bovine insulin from apical to basolateral chamber did not damage the monolayer (Fig. 3a). The total amount of bovine insulin transported through the monolayer was directly proportional to the dose loaded in the apical chamber (Fig. 3b). A cumulative permeation of 1.5±0.8 µg, 3.5±0.7 µg, 13.5±4.0 µg, and 24.9±5.0 µg was observed at the loading concentrations for 0.05, 0.15, 0.6, and 1 mg/well respectively and was dose-dependent (*r* ^2^: 0.99). These numbers translate into a cumulative percent transport of approximately 2.5–3.0% of the loaded dose, which confirms poor permeability of macromolecular peptides across the monolayer. Calculated apparent permeability coefficients (P_app_) for bovine insulin were in the range of 4.5±0. 9×10^−6^ cm/s (1 mg) to 5.4±2.9×10^−6^ cm/s (0.05 mg) (Table 1).

**Figure 4:**
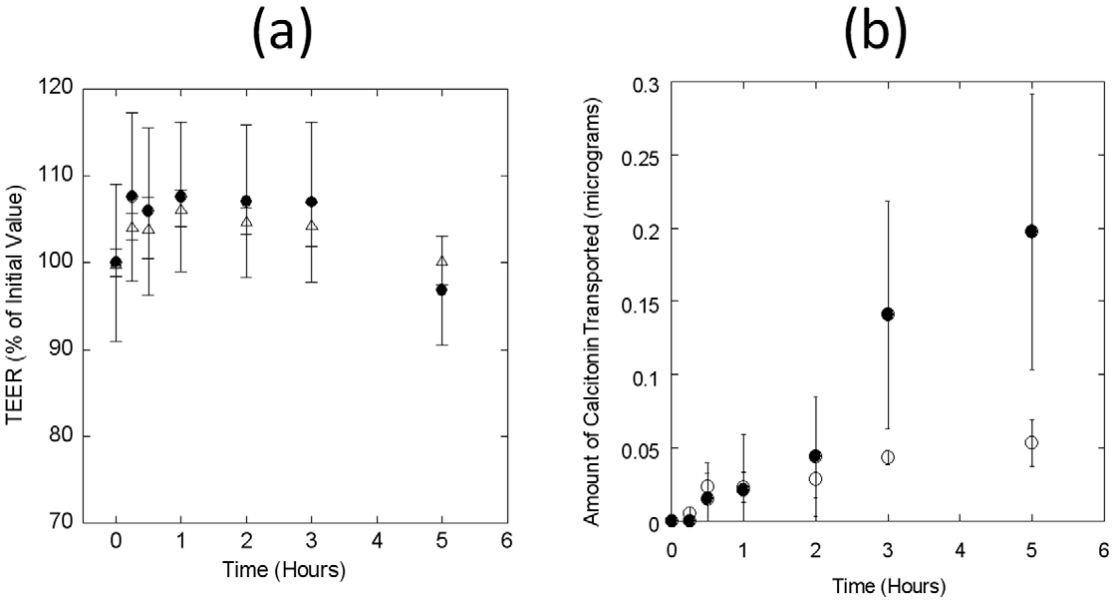
Transport of salmon Calcitonin (sCT) across Caco-2 monolayers. Permeation of salmon Calcitonin was measured at different loading concentrations. Apical-to-basolateral permeability was measured by analyzing the amount of sCT present in basolateral chamber at different time-points. TEER values were measured to validate integrity of the monolayers. sCT concentrations were measured by commercially available ELISA kits, as mentioned in the Methods section. (**a**) TEER values of Caco-2 monolayer at different sCT loading concentrations of 5.0 (circles) and 24.0 (triangles) µg/well. (**b**) Cumulative amount of calcitonin transported (µg) to the basolateral chamber at different time-points at different loading concentrations of 5.0 (open circles) and 24.0 (filled circles) µg/well. Data represent mean±SD (n = 3).

Permeability of salmon Calcitonin was determined at 2 different apical loading concentrations of 5 µg/well and 24 µg/well respectively. As in the case of insulin experiments, the TEER values did not drop during the course of the experiment (Fig. 4a). Transport of salmon Calcitonin was dose-dependent and total amount of the peptide transported increased in direct proportion to the loading concentration on the apical side. A total of 55±2 ng (5 µg loading), and 192±95 ng (24 µg loading) Calcitonin was transported to the basolateral side of the transwell system (Fig. 4b), which translates to 1.1±0.04% and 0.8±0.4% cumulative apical to basolateral permeation at 5 and 24 µg apical loading respectively (Table 1). The calculated P_app_ values for Calcitonin were in the range of 2.0±0.07×10^−6^ cm/s (Table 1).

**Figure 5:**
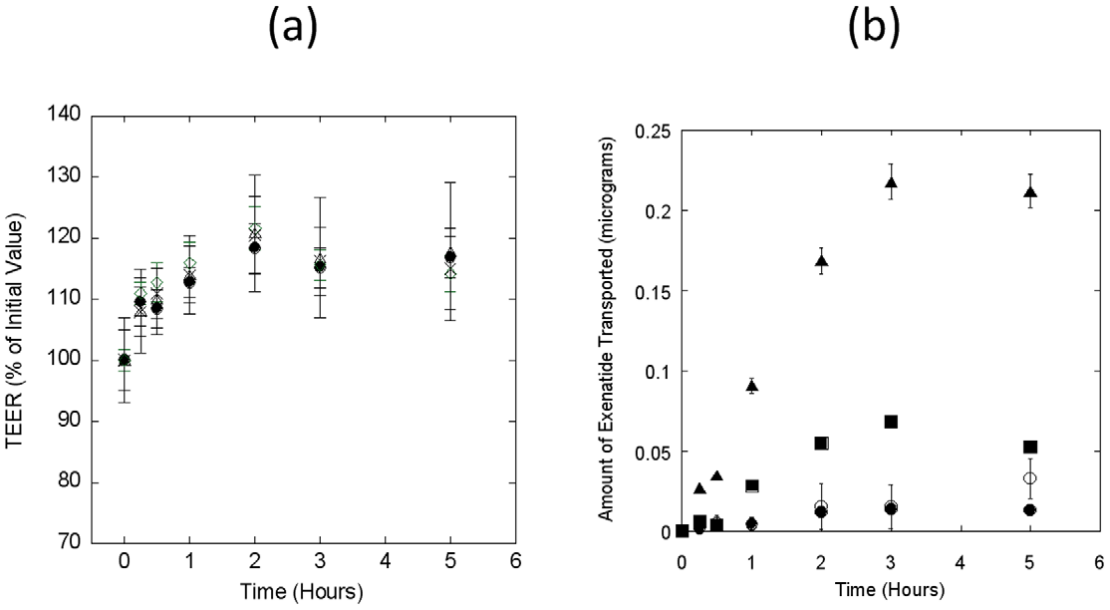
Transport of exenatide across Caco-2 monolayers. Apical-to-basolateral permeability of exenatide was measured at different apical loading. TEER values were determined to ensure monolayer integrity. (**a**) TEER values of Caco-2 monolayer following apical loading of different concentrations of exenatide at 0.3 (circles), 1.0 (triangles), 3.0 (diamonds), and 9.0 (crosses) µg/well. (**b**) Cumulative transport of exenatide (µg) to basolateral chamber during the experiment at different apical loading concentrations of 0.3 (filled circles), 1.0 (open circles), 3.0 (squares), and 9.0 (triangles) µg/well. Data represent mean±SD (n = 3).

Exposure of Caco-2 monolayers to different concentrations of exenatide also confirmed no damage to the monolayer's integrity (Fig. 5a). However, the transport of exenatide did not seem to be dose-dependent. Percent exenatide dose that transported through the Caco-2 monolayer decreased with increase in the loading concentration on the apical side (Fig. 5b). A total of 0.01±0.002 µg, 0.03±0.01 µg, 0.05±0.03 µg, and 0.2±0.1 µg was transported to the basolateral chambers for apical loading concentrations of 0.3, 1, 3, and 9 µg respectively (Fig. 5b). These numbers translate into a cumulative percent transport of 4.3±0.5%, 3.3±1.3%, 1.7±1.1%, and 2.4±1.2% respectively (Table 1). The highest P_app_ value of 7.8±0.9×10^−6^ cm/s was obtained with 0.3 µg/well apical loading. However, the P_app_ values decreased with increasing loading concentrations, and a value of 3.1±2.0×10^−6^ cm/s was obtained with 3 µg/well apical loading (Table 1).

## Discussion

This study validates the feasibility of short-term 3-day Caco-2 monolayers as a tool for assessing the bioavailability of several macromolecular and small molecular weight active pharmaceutical moieties, which are known to be very poorly bioavailable via oral route. Results from several *in situ* and *in vivo* studies are discussed herein to further support the observations. Three polypeptides with proven therapeutic potential were used in this study. Insulin is used for treatment of both type-1 and type-2 diabetes, and has been one of the most widely prescribed injectable therapeutic polypeptide 20]. Calcitonin is prescribed for treatment of post-menopausal osteoporosis and hypercalcemia, as a parenteral and nasal formulation 21]. The third polypeptide, exenatide is a glucagon-like peptide-1 (GLP-1) mimetic, and is FDA approved as an adjunctive subcutaneous therapy for treatment of type-2 diabetes mellitus 22]. Hence, this study provides valuable information regarding the use of short term 3-day Caco-2 monolayers as a model to predict oral absorption of therapeutic polypeptide.

The permeability profiles of FITC-insulin and sulforhodamine-B confirm the ability of short-term Caco-2 monolayers to study transport of macromolecules as well as small molecules across the intestinal epithelium. Apical-to-basolateral transport of both FITC-insulin and sulforhodamine-B correlated with the applied dose in the apical chamber. Both molecules permeated through the monolayer at similar rates for different loading concentrations through the experiments without damaging the monolayer. Pharmaceutical molecules may traverse through the intestinal epithelium via different pathways depending on their size and hydrophilicity. For example, macromolecules such as insulin may not permeate through the transcellular route due to their hydrophilic nature 23]. whereas small hydrophilic molecules such as sulforhodamine-B are ideal candidates for transcellular transport 7]. The 3-day short term Caco-2 culture model is capable of determining transport of both polypeptides and small molecules, and thus can be aptly used in studying intestinal transport of molecules permeating through different pathways.

To date, most of the *in vitro* permeation studies to predict *in vivo* behavior of peptides following oral administration have been performed on conventional 21-day Caco-2 culture 9], or in some cases with Caco-2/HT-29 co-cultures 24,25]. However, several reports suggest that conventional 21-day may not provide a rational assessment of potential oral bioavailability of the therapeutic molecules due to the large variations among permeation values reported in literature 13,17]. A number of studies have delineated permeation of bovine insulin through Caco-2 monolayers in a traditional 21-day transwell system 26–28]. However, the effects of a short-term 3-day Caco-2 culture system mimicking intestinal epithelium differentiation environment on insulin permeation through Caco-2 monolayers are relatively unknown. At the same time, a very limited number of studies have investigated transport of salmon Calcitonin 29,30] and exenatide across Caco-2 monolayers so as to predict their oral absorption.

Several published studies have reported apical-to-basolateral permeability values for insulin in the range from 1.6±0.4×10^−9^ cm/sec 31] to 0.5±0.3×10^−6^ cm/sec 32] at different apical chamber loading concentrations on a conventional 21-day monolayer formation. This corresponds to a 500-fold difference. At the same time, these values do not correlate with the actual permeability values of the intestinal tissues. For example, Woitski *et al.* reported insulin permeability through the rat intestinal mucosa to be of the order of 7.4±1.2×10^−6^ cm/sec using freshly excised jejunum with Ussing chamber model 33], which is significantly higher than the reported permeability values for insulin in Caco-2 systems. At the same time, our experiments with short-term 3-day Caco-2 co-culture revealed permeability coefficients for insulin to be 4.5±0.9×10^−6^ cm/sec at 1 mg apical loading, which is close to the reported intestinal permeability of insulin.

Similarly, the permeability values reported for salmon calcitonin across conventional 21-day Caco-2 monolayers in literature range from 1.4±0.3×10^−7^ cm/sec 34] to 8.5±2.3×10^−6^ cm/sec 35]. Sinko *et al.* reported calcitonin permeability values through rat jejunum to be 1.9±0.5×10^−6^ cm/sec 36], which correlates well with the permeability coefficients values of 2.0±0.07×10^−6^ cm/sec obtained with 3-day Caco-2 culture reported here.

Being a relatively newer therapeutic polypeptide, there are no reported studies for Caco-2 permeability of exenatide, a GLP-1 analog, which is approved as subcutaneous adjunctive therapy for type-2 diabetes. In a recent study, Youn *et al.* reported Caco-2 permeability of GLP-1 analog to be 0.5±0.2×10^−7^ cm/sec 37]. The non-linear dependence of exenatide transport on concentration suggests that unlike insulin and calcitonin, a saturable transport mechanism may exist for exenatide, which hinders higher amounts of the peptide passing through the epithelial membrane at higher concentrations of exposure. Several reports have suggested a dynamic influence of structural features of peptides on their biomembrane permeability 38,39]. Several reports have suggested presence of a saturable peptide transport in Caco-2 monolayer due to presence of either a concentration gradient, or due to interaction with the active peptide transporters in the monolayer such as Peptide Transporter 1 (PePT1) 40]. Although majority of epithelial transport of most polypeptides and proteins occurs via passive diffusion through aqueous-filled tight junctions 41], a significant amount also gets transported via active peptide transporter pathway. This saturable, apically polarized transport system in Caco-2 cells for peptides hinders apical to basolateral flux, enhances basolateral to apical flux, shows substrate specificity, and can get saturated by presence of various physiochemical stimuli 42]. Literature suggests that some surfactants and P-glycoprotein inhibitors can potentially be used to increase transepitheilal uptake and transport of various pharmaceutical moieties 43]. As exenatide is a relatively new therapeutic macromolecule, further investigations are required to establish its epithelial transport kinetics.

There may be several reasons for the inconsistent results reported related to permeability of peptides in literature, one of the major ones being the 21-day culture not physiologically mimicking the intestinal environment. The conventional Caco-2 monolayers are grown with growth medium supplementation with undefined animal serum and for extended culture periods to obtain the differentiated phenotype of intestinal absorptive cells (enterocytes), which takes about 3–4 weeks to develop. However, due to unnatural growth conditions, several phenotypic differences are observed with slight variations in the protocols. At the same time, the significantly higher TEER values reported for a tight junction 21-day culture are significantly higher (∼300 Ω.cm^2^) as compared to TEER values for human intestine (∼50–100 Ω.cm^2^) 14], which indicate the presence of unphysiologically tight pores. On the other hand, short-term 3-day Caco-2 monolayers yield moderate TEER values (∼100–150 Ω.cm^2^). The short-term 3-day Caco-2 monolayers are grown in an environment which mimics intestinal differentiation environment so as to provide enterocyte-like monolayer with barrier functions, thus physiologically mimicking the human intestinal epithelium. These monolayers are grown in a serum-free growth medium with supplements such as butyric acid, hormones, growth factors, and other metabolites, which are reported to regulate the differentiation of intestinal epithelial cells *in-vivo* 18,44–46]. At the same time, the 3-day Caco-2 monolayer systems have been tested for the presence of various physiological markers and enzymatic activity. As known, the conventional Caco-2 monolayers express a lot of peptidases both cell surface associated and intracellular including Dipeptidyl peptidase IV (DPP-IV), aminopeptidase N, aminopeptidase W, and alkaline phosphatase to name a few 47]. Optimum expression of these enzymes is vital for accurate prediction of macromolecular transport via through intestinal epithelium owing to extensive enzymatic degradation in gastrointestinal tract. 3-day Caco-2 monolayers were found to have no significant differences with 21-day Caco-2 systems in terms of peptidase levels (cell surface and intracellular) with comparable differentiation enzyme marker and P-glycoprotein activities 16].

The results presented here confirm the merits of a 3 day Caco-2 culture for studying permeation across the intestine over the conventional 21-day cultures. The data suggest that the permeability values obtained with 3-day monolayers correlate better with the intestinal permeability values, thus establishing 3-day Caco-2 culture as a viable alternative to current 21-day Caco-2 culture. The reported data also act as a bridge among the otherwise inconsistent permeability data published in a variety of studies. With additional studies focused on correlating the data with *in-vivo* absorption, short-term Caco-2 cultures can be used more efficiently for high-throughput screening of lead compounds or formulations 48,49]. This is the first study to test the 3-day Caco-2 system for macromolecules. Since the development of 21-day Caco-2 system, the pharmaceutical drug development has completely been overhauled with immense need for a rapid high throughput screening method for predicting oral absorption of investigational therapeutic macromolecules. Once validated, the 3-day system can well be developed as an in-house system in various high throughput screening laboratories both in academia and industry, which will further reduce the operational costs making it a logistically more beneficial approach, while providing outstanding correlation with in vivo data.
